# Medication-Related Osteonecrosis of the Jaw in Dental Practice: A Retrospective Analysis of Data from the Milan Cohort

**DOI:** 10.3390/dj10050089

**Published:** 2022-05-19

**Authors:** Cristina Mirelli, Sonia Marino, Andrea Bovio, Sara Pederielli, Cristina Dall’Agnola, Aldo Bruno Gianni, Roberto Biagi

**Affiliations:** 1 Department of Biomedical Surgical and Dental Sciences, School of Dentistry, University of Milan, 20122 Milan, Italy; cristina.mirelli@unimi.it (C.M.); andrea.bovio@unimi.it (A.B.); sara.pederielli@gmail.com (S.P.); cristina.dallagnola@unimi.it (C.D.); aldo.gianni@unimi.it (A.B.G.); roberto.biagi@unimi.it (R.B.); 2 UOC di Chirurgia Maxillo-Facciale e Odontostomatologia, Fondazione IRCCS Cà Granda Ospedale Maggiore Policlinico, Via della Commenda 10, 20122 Milan, Italy

**Keywords:** bisphosphonate, denosumab, osteonecrosis, MRONJ, Antiresorptive Agent-Related Osteonecrosis of the Jaw (ARONJ), osteoporosis, risk factor

## Abstract

A retrospective analysis was performed with the aim of understanding whether the risk factors showed in the literature for medication-related osteonecrosis of the jaws (MRONJ) in cancer patients are also relevant in osteoporotic patients taking antiresorptive drugs (ARDs). Data were retrospectively pooled from health records of patients on ARDs who requested a dental visit between January 2006 and April 2020 in the Dental Unit at Fondazione Ca’ Granda IRCCS Ospedale Maggiore Policlinico, University of Milan. A total of 434 patients were included. The following variables were collected: sex, age, smoking habit, type of ARD, duration of treatment, route of administration, therapeutic indication, concurrent systemic therapies and pathologies. Statistical analysis confirmed the relevance of chemotherapy, smoking, and immunosuppressive drugs as risk factors. In addition, a higher frequency of MRONJ in osteoporotic patients was reported in our cohort in association with an immunodeficiency disorder of variable origin. In conclusion, the identification of individual risk-profile before dental treatments is crucial for prevention. Anamnesis should include main risk factors, such as immunosuppression, dental extractions, smoking, trauma, and poor dental health. Nevertheless, our suggestion for dental professionals is to conduct a complete medical history of patients who mention long-term per oral therapies with ARDs for osteoporosis. Osteoporotic, as well as cancer patients, may also benefit from periodic monitoring of the ARDs therapy in order to prevent MRONJ.

## 1. Introduction

Osteonecrosis of the jaws (ONJ) associated with the use of drugs was first reported in the literature by Marx in association with bisphosphonates (BF) [1]. It has therefore been called bisphosphonate-related osteonecrosis of the jaws (BRONJ), and is described as an area of bone exposure in the maxillofacial and intraoral region which shows signs of delayed or slow healing. The lesion may be asymptomatic or sore with swelling, purulent discharge, tooth involvement or mobility and paresthesia [2,3,4]. This definition has undergone several alterations. After the marketing of new antiresorptive drugs (ARDs) other than bisphosphonates, this complication changed its name from bisphosphonate-related osteonecrosis of the jaws (BRONJ) to medications-related osteonecrosis of the jaws (MRONJ). In 2014, the American Association of Oral and Maxillofacial Surgeons (AAOMS) updated the original 2007 definition [5]. The new description also includes cases of osteonecrosis with intra- or extraoral fistulas without bone exposure in patients without metastases or previous radiotherapy in the head and neck region. A more detailed staging is described in the most recent AAOMS Position Paper 2022 Update [6].

ARDs are currently among the most prescribed drugs in the world and represent the gold standard for treatment and/or prevention of multiple myeloma, bone metastases, malignant hypercalcemia (tumor-induced), some solid tumors (e.g., prostate cancer, mammary carcinoma), as well as osteoporosis and some bone disorders such as Paget’s disease and osteogenesis imperfecta. MRONJ rate greatly differs between cancer and osteoporotic patients. In the first case, ARDs are taken intravenously (i.v.) and incidence varies between 1.0 and 8.0%, while in the second case, the therapy is taken perorally (p.o.) and MRONJ occurrence varies between 0.2 and 0.4% [7,8].

Risk factors for MRONJ have been extensively investigated in patients with cancer, as necrosis often occurs after the use of ARDs for prevention of bone metastases [9,10,11]. The population treated with ARDs for other reasons has rarely been evaluated as the prevalence of MRONJ among patients treated for osteoporosis is low and varies in percentage between studies [6]. Nevertheless, it appears to be extremely important to examine the general population as the prescription of ARDs for non-oncological conditions is relentlessly increasing. These patients are the majority of outpatients and private patients that may be at an increased risk of osteonecrosis, representing a new challenge for dental practitioners [12,13,14,15,16,17,18,19,20].

At present, prevention is still the most significant approach for protecting the oral health of patients treated with ARDs since none of the available therapies proved to be effective in treating MRONJ, which remains a disabling complication with pejorative development [21]. For this reason, general practitioners (GPs) or other specialists who find the need to prescribe a therapy with BF and/or with so-called biological target drugs (i.e., bevacizumab, sunitinib, sorafenib), or with other ARDs (i.e., denosumab), must inform patients of the possibility of an assistance path.

In this perspective, the “Progetto Bifosfonati—Project Bisphosphates” (PB) was set up at the Fondazione Ca’ Granda IRCCS Ospedale Maggiore Policlinico in Milan to assist patients who are about to start or are currently undergoing therapy with ARDs. The goal of PB is to deliver a specialized dental service to all patients, including those from various hospital wards and private clinics. The influx of such a variety of patients allowed us to evaluate each patient via standardized medical history and data collection. 

The aim of the study was to carry out a retrospective statistical analysis on data collected from the PB regarding the incidence of MRONJ, and to investigate if known systemic risk factors of MRONJ have the same relevance in cancer and osteoporotic patients taking ARDs. 

## 2. Materials and Methods

**Figure 1:**
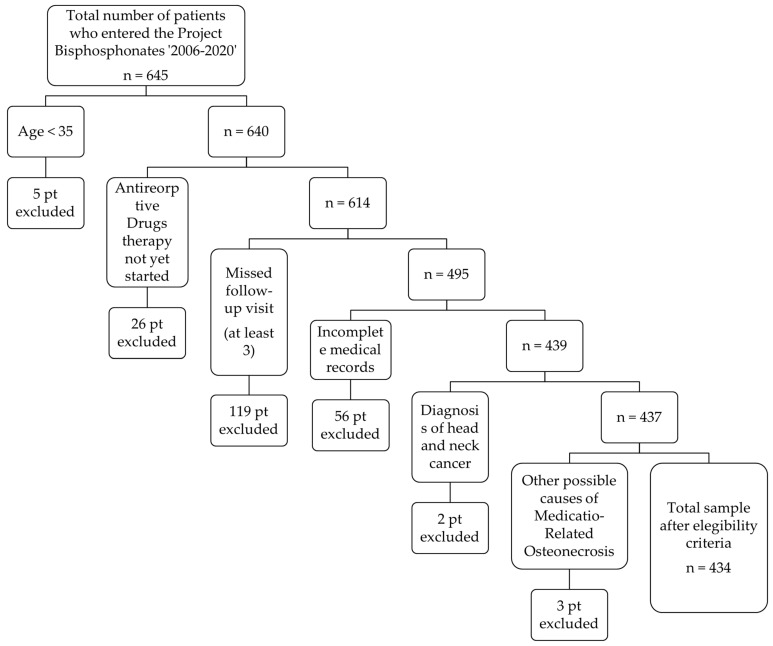
Workflow of patient selection.

This study is a single center retrospective epidemiological research on medical records collected in the PB. Data was collected from the patients’ clinical records according to selection criteria exemplified in Figure 1.

Since 2006, the PB has offered first visit service to anyone about to start taking ARDs or already undergoing therapy, whether or not osteonecrosis is present. The aim of PB is to create a personalized treatment path based on counselling, monitoring and prevention.

Upon entering the Unit of Restorative Dentistry, every patient is asked to grant consent to the medical treatment and data processing. PB uses a customized medical record which includes an in-depth medical and dental history. Particular attention is paid to data regarding risk factors for MRONJ, therapy in progress and patient lifestyle.

For each patient, the following parameters were collected: sex, age, smoking habits, clinical motivation for taking ARD, type of ARD taken, duration of ARDs therapy, other drugs taken (antidepressants, chemotherapy, corticosteroids, levothyroxine, immunosuppressants, proton pump inhibitors (PPIs), metformin, strontium ranelate, hormonal replacement therapy (HRT) and antiangiogenic therapy), concurrent pathologies and pre-existing medical conditions (anemia, diabetes, hypo- or hyperthyroidism, and autoimmune pathologies). Cases of MRONJ presenting clinical or radiographic evidence were identified following the criteria defined by AAOMS 2014 [5]. 

The sample was divided into different subgroups with specific characteristics in order to compare the occurrences of MRONJ. Firstly, sex and age were considered: patients were divided into five age groups (35–49; 50–59; 60–69; 70–79; >79). Secondly, groups based on the therapy were selected; the type of drug, the number of ARDs, and the duration of the treatment were taken into consideration. As far as the duration of the therapy is concerned, patients were divided into nine classes (from 0 to 10, at two-year intervals; from 10 to 30, at five-year intervals). Then, the sample was divided into three sorted groups depending on indication to treatment with ARDs: Oncological group (G1), osteoporotic group (G2), and a mixed group composed of patients with indication of both cancer and osteoporosis (G3). Based on a combination of parameters, increasingly specific and less numerous subgroups were selected. 

The Cochran–Mantel–Haenszel Test was used as mass test, where applicable, otherwise the Fisher exact test was repeated on the different strata. Where mass test gave relevant results, the odds ratio (OR) was used to find conditions with significant differences between groups. The significance level was chosen to be 0.05 and, where necessary, post-hoc correction was applied using the Bonferroni method. While analyzing the correlation between age and prescribed drug, the Kruskal–Wallis test was used.

Furthermore, an electronic search of Medline (PubMed), Cochrane, SSCI (Social Citation Index), and SCI (Science Citation Index) databases from 1990 to present was performed to collect data on the epidemiology of bisphosphonates. Results of this research were used to compare our findings. The following search words were used: antiresorptive-related osteonecrosis of the jaw (ARONJ), MRONJ, bisphosphonates, denosumab, ARDs, osteoporosis, bone metastasis, risk profile and preventive protocols.

## 3. Results

Below are the results of the analyses performed to describe the characteristics of our sample in relation to noteworthy conditions and presumed risk factors for MRONJ. Out of 434 patients included in this study, 34 patients developed MRONJ. Among all visited patients, only eight referred to us before starting ARDs treatment. Sample characteristics are showed in Table 1 and Table 2. 

### 3.1. Incidence of MRONJ by Age

**Figure 2:**
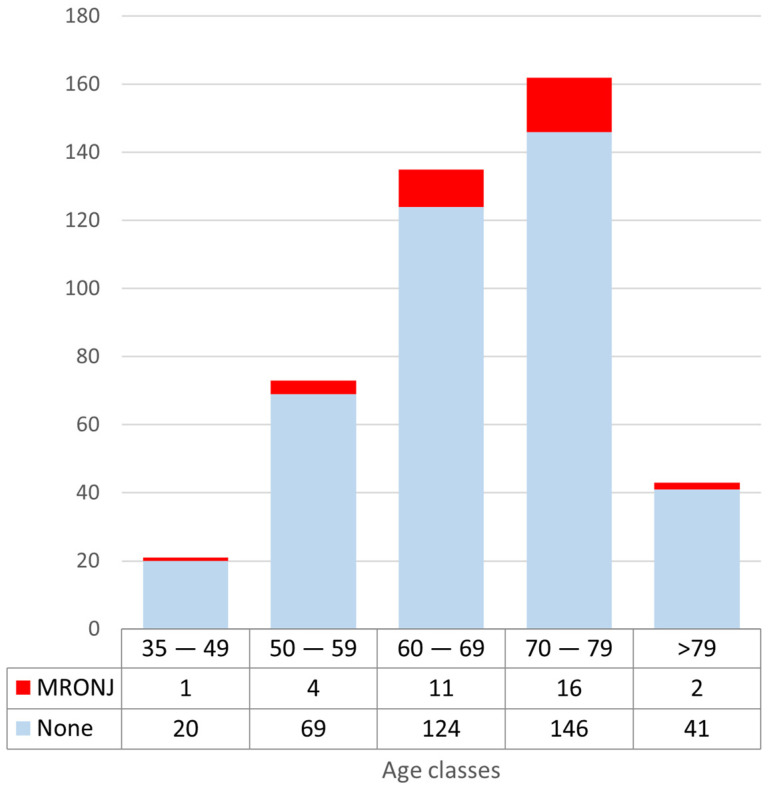
MRONJ incidence by age groups.

In order to analyze the incidence of MRONJ by age, the cohort was divided into five age groups (35–49; 50–59; 60–69; 70–79; >79). Even though the incidence of MRONJ is higher between 60–69 and 70–79, no correlation was statistically significant among age groups (Figure 2), not even when the additional parameter of concurrent pathologies treated with ARD (see point 3.4) was introduced.

### 3.2. Incidence of MRONJ by Gender

MRONJ occurred in 5 males and 29 females, for an OR (F:M) = 1.47; however, there was no statistically significant difference in the number of cases between the two groups.

### 3.3. Incidence of MRONJ by Duration of Treatment with ARD

According to duration of treatment, the sample was divided into nine classes (from 0 to 10, at two-year intervals; from 10 to 30, at five-year intervals), and a Fisher test was performed. This subdivision is an arbitrary choice, made to populate the groups in a more homogeneous way.

**Figure 3:**
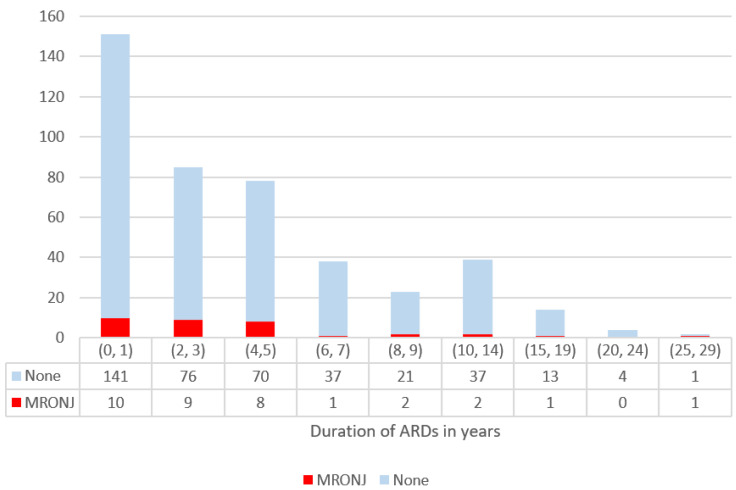
Detail of stratification by age.

The comparison among groups, which were divided based on duration of the therapy, did not yield statistically significant differences (*p*-value = 0.42) (Figure 3).

### 3.4. Association with Known Pathologies

The analysis showed that 79 patients took ARDs for cancer (G1), 298 for osteoporosis (G2), while 57 presented both diseases (G3). The most common oncological diseases are listed in order of frequency: breast cancer (68), multiple myeloma (22), prostate cancer (12), cutaneous melanoma (4) (Table 2). 

G1 had a higher incidence of osteonecrosis (15.8%). According to the z-test, this difference was statistically significant when compared to patients in G2 and G3 (*p*-value = 0.03).

The number of MRONJ cases did not seem to be affected by the type of cancer detected in each patient: breast cancer (14/68); myeloid cancer (6/22); prostate cancer (1/12); skin cancer (1/4). In fact, these differences are not statistically significant according to the two-tailed Fisher test (*p*-value = 0.58).

### 3.5. Incidence of MRONJ by Type of ARD

**Figure 4:**
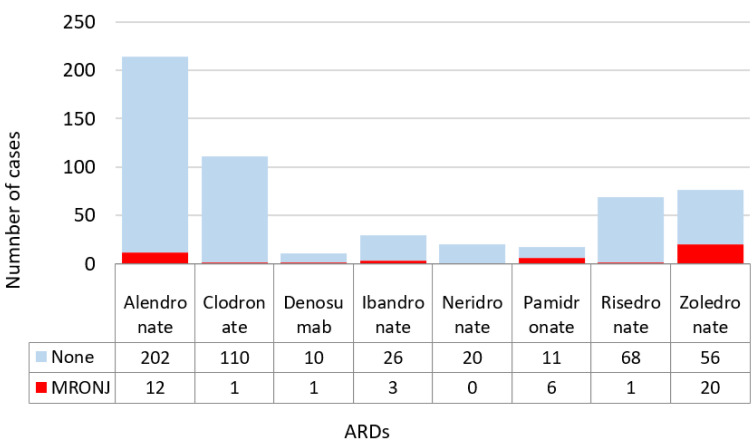
MRONJ incidence by ARDs taken; Figure refers to the total number of drugs taken.

**Figure 5:**
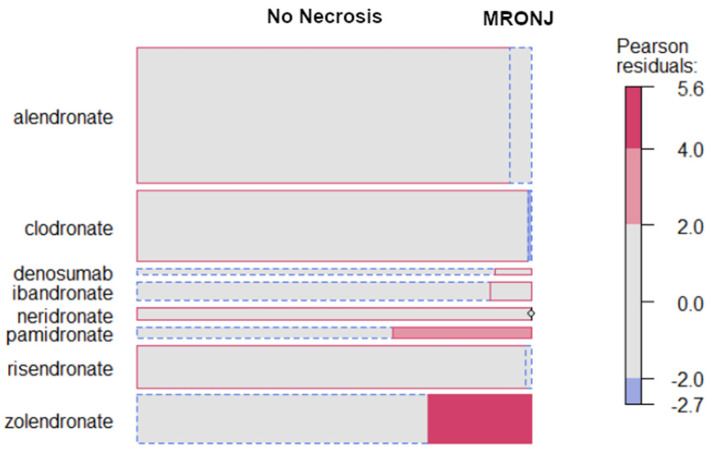
Pearson residuals express difference from mean (expected) in sigma units. Its clinical meaning is a shortcoming of expected cases in patients treated with clodronate compared to the other ARDs.

Many patients took a combination of more than one drug. The following results were therefore obtained considering the sample size equal to the total number of drugs taken. The incidence of necrosis is significantly associated with the type of ARD taken (Fisher test, *p*-value = 0.0005), as shown in Figure 4. Figure 5 shows an excess of cases in association with consumption of zoledronate, pamidronate and, to a lesser extent, denosumab.

As far as clodronate is concerned, fewer cases than expected were found. Results also showed that the probability of developing necrosis increased when two ARDs were taken together or subsequently. The number of cases of MRONJ was significantly higher in the group of patients who took two drugs (OR (1: 2) = 1.67, *p*-value = 0.0102).

### 3.6. Incidence of MRONJ by Route of Administration

**Figure 6:**
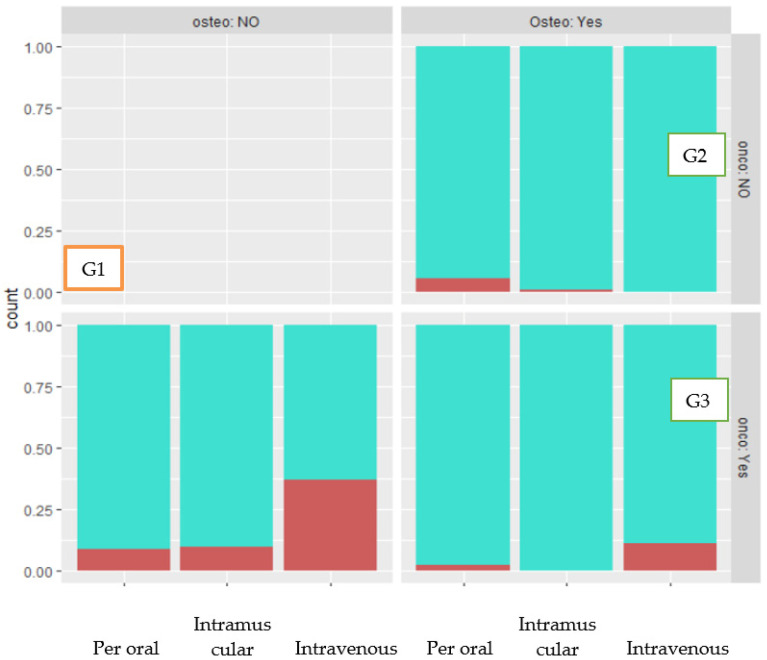
Relative incidence of MRONJ in G1, G2, and G3 by route of administration.

According to Fisher’s test, it was found that i.v. ARDs use in combination with cancer resulted in a significantly higher rate of necrosis (24 patients) compared to all other cases (*p*-value = 0.01482) (Figure 6).

### 3.7. Incidence of MRONJ in Patients with Other Concurrent Diseases

In this analysis, the following diseases were considered: anemia, diabetes, hypo- and hyperthyroidism and autoimmune diseases. Many patients had a combination of multiple diseases; the sample size therefore corresponds to the total number of diseases present in the cohort. This sample was divided into specific groups based on pathology. The incidence of MRONJ in each class was compared to the incidence in patients without concurrent diseases. No statistically significant difference was reported.

### 3.8. Incidence of MRONJ in Patients Receiving Other Drugs

In this analysis, the following drugs were taken into consideration: antidepressants (D1), chemotherapy (D2), corticosteroids (D3), sodium levothyroxine (D4), immunosuppressants (D5), proton pump inhibitors (PPI) (D6), metformin (D7), strontium ranelate (D8), thalidomide (D9), HRT, methimazole (D10). Many patients took multiple drugs; the sample size therefore corresponds to the total number of medications taken plus the number of patients not taking any. The sample was stratified into classes based on the above-mentioned drugs, in addition to a group for patients not taking any other medications besides ARDs.

**Figure 7:**
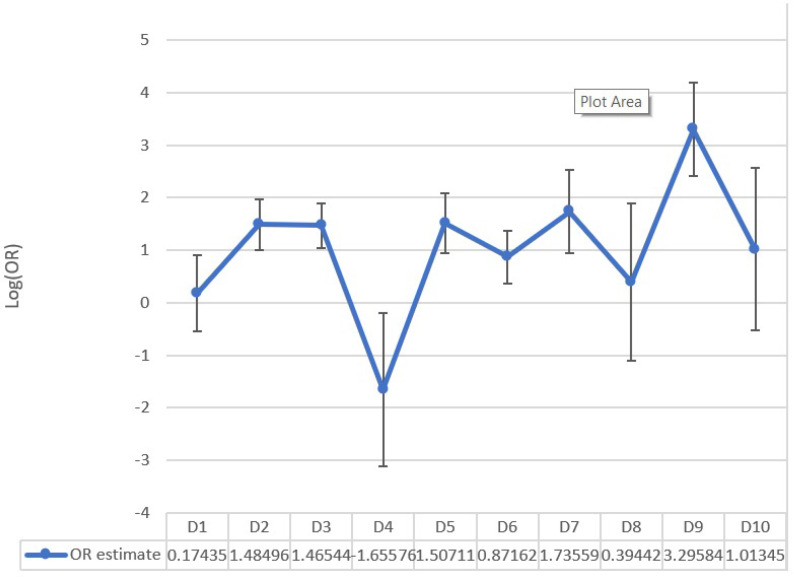
A summary of odds ratio log for each class of drugs vs. no drugs.

The incidence of necrosis was compared between groups receiving the drug and the group without any intake. The comparison of the ORs is shown in Figure 7.

A statistically significant difference for OR appeared for the following classes of drugs after Bonferroni correction: chemotherapy (corrected *p*-value = 0.019), corticosteroids (corrected *p*-value = 0.004), thalidomide (corrected *p*-value = 0.002). The number of cases of necrosis for each type of ARD in relation to the intake of all other drugs was included in a further analysis, which highlighted evidence that certain drug combinations are related to an increased incidence of MRONJ.

Corticosteroids and thalidomide are associated with MRONJ, especially in combination with zoledronate. After verifying the association between immunosuppressants and zolendronate, we counted six patients taking corticosteroid and zoledronate, and four patients taking antiangiogenics (sunitib and thalidomide). Furthermore, we reported eight patients taking alendronate with MRONJ, and four of them were on a long-term therapy with immunosuppressant drugs like corticosteroid or leflunomide. A second analysis was performed comparing groups of patients taking ARDs in combination with other drugs to the groups of patients who received the same ARD but were not taking any other drugs.

These data, tested for significance in OR, are not confirmed after the post-hoc correction. Due to the considerable number of combinations, we have a small number of patients with which to verify these specific events.

Considering only G2, there seems to be a higher incidence of MRONJ in the case of association between alendronate and immunosuppressants. The significance, however, is not confirmed after the post-hoc correction.

A total of 43 patients were taking or had taken hormonal replacement therapy (HRT) and at least one ARD at the same time. Out of 34 patients reporting MRONJ, five patients were on HRT, resulting in OR = 1.64. According to the z-test, this difference was not statistically significant.

### 3.9. Incidence of MRONJ in Relation to Smoking

MRONJ occurred in 29 smokers (S) and 5 non-smokers (N), for an OR (S: N) = 0.83. This difference in the number of cases between the two groups was marginally significant, according to the z-test (*p*-value = 0.04636).

Table 3 summarizes the statistical results of our research.

## 4. Discussion

MRONJ is a relatively recent phenomenon [1] and is widely discussed and well known to the scientific community. Nevertheless, scientific literature suggests it is a poorly understood subject by doctors and dentists (17.3–40%) [22,23,24]. A 2018 canvass report [25], for example, shows that 84.6% of dentists did not recognize any trade names of ARDs. Similarly, knowledge concerning the risk factors, including concomitant pathologies and risk factors, is between 50 and 56% among doctors [26,27]. It is no coincidence that a 2021 online survey about the awareness of MRONJ among dentists from Central Europe showed a considerable lack of scientific knowledge about this clinical complication and its management [28]. Our statistical analysis confirmed that oncological patients are much more at risk of developing MRONJ. Concerning additional risk factors, we found significant results regarding smoking, chemotherapy and corticosteroids, which in combination with ARDs, seem to favor MRONJ in both oncological (G1) and osteoporotic (G2) groups.

In the discussion, we will compare results of our retrospective analysis from the PB cohort with results found in scientific literature.

In our cohort, the mean age of patients who did not develop MRONJ was 68.52, while for those who developed the complication it was 70.03. The latter is slightly higher in comparison to Vereb et al. (66.80), but overlapping with Rogers et al. (70, range 61–77) [15,29]. No statistically significant difference was found for the incidence of osteonecrosis between age groups in our study. On the contrary, Vatshevanos et al. reported the correlation between age and osteonecrosis to be valid (r = 0.187; *p* < 0.001) [30].

Our study confirmed the prevalence of MRONJ in female subjects with a male/female ratio of 1: 7.2. Vereb et al. recently found a ratio of 1: 2.1, and Owosho et al. of 1: 1.3, while Pazianas et al. found a ratio that seems similar to ours, ranging from 1: 8 to 1: 5.8 [29,31,32]. This would appear to be due to the very nature of the sample, mixed in our case (with both oncological and osteoporotic patients) and purely oncological in the aforementioned two. The higher prevalence of cases in women is probably due to a higher incidence of diseases for which ARD drugs are prescribed [5].

Menopause is one of the unchangeable risk factors for osteoporosis, which is why postmenopausal women are particularly at high risk of developing osteoporosis from the age of 50. It is not a coincidence that we find an incidence of 10.60% in males and 7.40% in females. In fact, the male sample is almost entirely constituted by oncological patients, while the female one more often comprehends an increased number of patients affected by metabolic pathologies. The diagnosis of osteoporosis, and therefore the administration of ARD drugs, is constantly increasing [13]. This phenomenon concerns doctors and dentists, especially because of the high number of these patients and therefore their frequency in daily clinical practice [8].

The duration of ARDs therapy as a risk factor for MRONJ is a controversial discussion in international literature. In a 2009 review, Palaska et al. found a time to onset (TTO) of 1.8 years for zoledronate and 4.6 years for alendronate [33]. Other authors reported a TTO between 1.2–3 years for alendronate and 2.5–5.9 years for zoledronate [34,35,36]. 

A recent multicenter study, based on TTO, found an average TTO for the entire cohort of 3.2 years, but specifically 2.2 years for zoledronate and 6 years for alendronate [37]. Lazarovici et al. pointed out that, for higher potency drugs, the increase in the duration of therapy is highly associated with the development of MRONJ [34]. 

In our study, the increase in osteonecrosis cases related to the duration of therapy with ARDS was not statistically significant.

The incidence of MRONJ in patients with osteoporosis associated with intake of ARDs p.o. in our cohort was higher than expected (5.08%). In the literature, the incidence of MRONJ in osteoporotic patients has rarely been assessed and was found to be about 0.01–2.27% [5,38,39]. In 2007, Pazianas et al. stressed the high number of oral bisphosphonates prescriptions to patients who visited for osteoporosis. About 73% were prescribed ARDs for prevention only, ignoring the consequences that these drugs may bring in the presence of known risk factors [32]. A 2013 German study showed that the risk of MRONJ was underestimated in osteoporotic patients. In a correspondence survey of 107 dental practices, 37 cases of MRONJ were reported, of which 37.4% related to cancer and 62.6% to osteoporosis [38]. This emphasizes the problem of a lack of literature on the risk of MRONJ for this category of patients, which in relation to their size can be a growing concern [40].

In our survey, we recorded a prevalence of 26.32% for zoledronate. In the literature, the incidence of MRONJ in patients with zoledronic acid therapy varies within a wide range: 2.90–38.00%, which overlaps with our data [35,41,42,43,44]. 

Our survey also found a high incidence of MRONJ for pamidronate (35.29%), higher than the average in literature (4.00–18.00%) [31,35,41,42,44].

Alendronate is the most frequently administered drug among ARDs [32,45]. Indeed, 40.8% of patients in our sample were prescribed alendronate. Sedghizadeh et al. reported that the prevalence of MRONJ was about 4% in their sample treated with alendronate, and in our survey it was comparable (5.08%) [46]. This incidence can be explained by the remarkably high frequency with which alendronate is prescribed, as well as by its dosage, potency, half-life and absorption factors [2]. Alendronate is also a bisphosphonate containing nitrogen, known to pose a higher risk to MRONJ than nitrogen-free-bisphosphonates [47]. When compared with the percentage of patients who take clodronate (96), the numerical gap of incidence of MRONJ is conspicuous (5.08% vs. 1.04%).

Moreover, the role of corticosteroids and immunosuppressants taken for autoimmune diseases may require further investigation: in the research of Bendilayi et al., these therapies turned out to be associated to a greater inhibition of bone remodeling if associated with oral ARDs [47]. Chiu et al. investigated a sample of patients only taking alendronate: the twelve cases of osteonecrosis were found in association with long-term use of corticosteroid [12]. In the study by Pazianas et al., at the time of diagnosis, 20% of patients with MRONJ took corticosteroid at the same time as oral antiresorptive drugs [32]. A 2009 experimental study in mice showed that concomitant intake of ARDs and corticosteroid produced hard tissue changes like those occurring in MRONJ lesions [48]. However, the association between corticosteroid therapy and necrotic lesions remains highly controversial, mainly due to a lack of specific statistical analysis. In 2007, Jadu et al. found a statistically significant value between the simultaneous administration of prednisone and ARDs and the appearance of ONJ (*p* = 0.014) [49]. Later works in the literature have shown the dangerousness of the association between corticosteroid and bisphosphonate [50,51]. In a 2017 report, Wong et al. stressed the importance of this correlation, mainly regarding the duration of corticosteroid therapy [52]. In our survey, we found a possible correlation between the contextual administration of alendronate and immunosuppressants and the development of MRONJ (*p* = 0.0224). However, it does not pass the post-hoc test on the critical value: the main problem is the relatively low incidence that would require a larger sample. According to our analysis, the administration of antiangiogenic drugs in combination with ARDs increases the risk of development of MRONJ too (*p* = 0.0002). However, little is known about the incidence and prevalence of MRONJ in patients with exclusive antiangiogenic therapy [53], and future analysis should be aimed in this direction.

Our data confirm the chemotherapeutic drug association with MRONJ, which was first reported in literature by Marx [1], and has been proven by several research since then [54,55,56,57,58].

As for the additional risk factors considered, a cohort study examined the presence of potential risk factors for MRONJ in a population of 60,000 individuals in need of surgical treatment and found that the risk was greater in patients with rheumatoid disease and use of PPI, independently associated with MRONJ [59]. We analyzed the data aiming to investigate the possible correlation with PPI since it was associated with a fair number of cases of MRONJ, combined with different types of ARDs. In our report, a significance analysis was performed for the incidence of MRONJ in the group of patients treated with PPI, considering the intake of the other additional drugs. However, it was found that no PPI case significantly increased the likelihood of developing MRONJ among patients taking other drugs. This suggests that the number of necrotic events in patients treated with PPI is a coincidence based on other risk factors (*p* = 0.3442). Lastly, the habit of smoking appears to be an important risk factor for MRONJ in our analysis (ORs (S: N) = 0.83). As is known, smoking increases vasoconstriction, and this can lead to an ischemic situation, favoring ONJ according to a study by Izzotti conducted in 2013 [60]. This association had already been reported in the literature by Nisi et al. (*p* = 0.04) and by Quispe et al. (*p* = 0.049) [50,61]. Other authors instead found no correlation through statistical analysis (*p* = 0.115) [30].

## 5. Strengths and Limitations

We present a statistical analysis of data collected form a diverse sample including all clinical cases sensitive to the administration of ARDs. This sample represents the common clinical case studies for dentists. Our results strengthen the knowledge of risk factors of MRONJ and are of help in daily practice. We selected a set of risk factors verified by previous studies. We also considered some secondary factors which themselves can change bone metabolism, and whose association is documented by less solid evidence. The link between all these risk factors and necrosis events was reported in the results and discussion. There are some limits to this research: it was conducted at a single center and on a limited sample. The statistical methods used are appropriate for interpreting the characteristics of the phenomenon, but a larger sample could give us a more representative image of reality. For this reason, we cannot make further considerations about the relationship between medical history and MRONJ without decreasing the reliability of our conclusions. We have highlighted the possible link between MRONJ and conditioning aspects of the medical history (for example of autoimmune diseases). However, due to the small sample size, we were unable to carry out specific analyses on the synergies and interactions between drugs. A multi-center study will allow us to progress in the analysis of these aspects. Moreover, we cannot exclude that the incidence rates for MRONJ that we reported were influenced by an upstream selection made by doctors or dentists who send patients to the PB to prevent the risk of MRONJ induced by ARDs. 

## 6. Conclusions

Our findings lead us to the conclusion that there is an underestimation of the risk of MRONJ associated with ARDs therapy in patients with dental problems. Bibliographical research confirmed a lack of awareness among dentists regarding osteonecrosis. Dentists should be trained to identify the high-risk profile for MRONJ of these patients and possibly refer them to highly specialized centers for consultation. 

Since MRONJ has a multifactorial etiology, patient care must be managed with a multidisciplinary approach. We encourage communication and collaboration between the different professionals involved in managing this complication. As a first step toward prevention, oncologists should recommend their patients to undergo a dental examination before starting treatment. 

With regards to specific subgroups of osteoporotic patients undergoing long-term p.o. therapies with ARDs, they are particularly vulnerable to side effects of corticosteroids. Doctors prescribing ARDs for osteoporosis should advise patients to inform their dentist about the ongoing therapy and schedule periodic visits to re-evaluate the ARDs treatment.

## Figures and Tables

**Table 1:** The sample consists of 387 females and 47 males, aged between 36 and 91, with an average age of 68.64. The subdivision into quartiles returned a median age of 69, with the first quartile 62, and the third quartile 76.

TOT Nr	434
Mean age	68.6
Median age	69
Age range	36–91
Interquartile range age	62–76
Sex	
Male	47
Female	387

**Table 2:** Indication for treatment with ARDs, main oncological diagnoses are listed.

	Indication to Treatment	Oncological Diagnosis
Osteoporosis	298	
Osteoporosis and oncological disease	57	136
Only oncological disease	79	
Breast cancer		68
Multiple myeloma		22
Prostate cancer		12
Lung cancer		8
Uterine cancer		6
Others		20

**Table 3:** A summary of results about MRONJ risk development considering the primary ARD taken and the main adjunctive factors.

Environmental Factors	Non-MRONJ Count	MRONJ Count	Statistically Significant Results
Smoking	69	5	0.04
Non-smoking	331	29	
Type of medications			
Alendronate	168	9	0.0306
Clodronate	95	1	Non-significant
Denosumab	5	1	Non-significant
Ibandronate	17	1	Non-significant
Neridronate	13	0	n.a.**
Pamidronate	10	4	Non-significant
Zoledronate	46	1	Non-significant
Risedronate	46	17	0.0333
Drugs			
Antidepressants	42	2	Non-significant
Chemotherapy	55	10	0.0019385 *
Corticosteroids	88	15	0.0004494 *
Levothyroxine	50	0	Non-significant
Immunosuppressants	26	4	Non-significant
Proton pump inhibitors (PPIs)	65	8	Non-significant
Metformin	10	2	Non-significant
Strontium ranelate	6	0	Non-significant
Thalidomide	5	3	0.0002010 *
Hormonal replacement therapy (HRT)	38	5	Non-significant

* According to Bonferroni post-hoc correction. ** MRONJ was never reported.

## Data Availability

The data presented in this study are available in this article.
